# Massive Open Online Courses on Health and Medicine: Will They Be Sustainable?

**DOI:** 10.2196/jmir.3798

**Published:** 2014-08-25

**Authors:** Kieran Walsh

**Affiliations:** ^1^BMJ LearningLondonUnited Kingdom

In this issue of JMIR, Liyanagunawardena and Williams have provided fascinating insight into the world of massive open online courses (MOOCs) as they have emerged over the past few years [1]. Their findings are clear: there is already a range of MOOCs available and they can be used for a variety of purposes in undergraduate medical education and continuous medical education for medical students, doctors, and health care professionals, with potential in health education amongst the general public.

These are interesting findings and may represent a significant “next step” in the provision of online learning in health care professional education. However, some would say that the past ten years have been as much about the hype of online learning as about the real outcomes that it can actually achieve [2]. This phenomenon is not peculiar to online learning—it happens with virtually all new media when they are initially introduced to education. In past few years however, there has been a shift in thinking about this new delivery mechanism of learning. Exponents of online learning no longer claim that it can do everything or that it will replace face-to-face education, rather, they are starting to think about how it can be used strategically and how its advantages can be adequately harnessed. Such advantages might include its flexibility or increased access to learners. This new and sober atmosphere with regard to online learning in medical education means that it is probably a good time for the medical education community to look at how MOOCs can be harnessed to deliver better education.

Certainly MOOCs satisfy many of the criteria that providers of medical education would like to achieve. They enable increased access, flexibility, and choice to the learner by offering learning at a time and place that suits the learner with substantial amounts of educational content. Another important component is that they are free to the learner. Free access is clearly important to many learners, but the current business model for the provision of MOOCs remains uncertain. Online learning is associated with significant costs, which cannot be ignored in the current economic environment [3]. How long will universities be able to make their content freely available throughout the world without undermining their basic business model, which involves charging learners for their courses? The answer to this question will likely help us draw conclusions as to whether MOOCs are just another passing technology fad or a sustainable long-term solution.
